# Beneficial effects of an intergenerational exercise intervention on health-related physical and psychosocial outcomes in Swiss preschool children and residential seniors: a clinical trial

**DOI:** 10.7717/peerj.11292

**Published:** 2021-04-27

**Authors:** Alice Minghetti, Lars Donath, Lukas Zahner, Henner Hanssen, Oliver Faude

**Affiliations:** 1 Department of Sport, Exercise and Health, University of Basel, Basel, Switzerland; 2 Department of Intervention Research in Exercise Training, German Sport University, Cologne, Germany

**Keywords:** Physical performance, Social-emotional skills, Functionality, Homes of the elderly, Mental health, Quality of life

## Abstract

**Background:**

Intergenerational exercise possesses the potential to becoming an innovative strategy for promoting physical activity in seniors and children. Although this approach has gained attraction within the last decade, controlled trials on physical and psychosocial effects have not been performed yet.

**Methods:**

Sixty-eight healthy preschool children (age: 4.9 y (SD 0.7)) and 47 residential seniors (age: 81.7 y (7.1)) participated in this five-armed intervention study. All participants were assigned to either an intergenerational (IG), peer (PG) or a control group (CON). Children were tested on gross motor skills (TGMD-2), jump performance and handgrip strength. Social-emotional skills questionnaires (KOMPIK) were assessed by kindergarten teachers. Seniors performed the Short Physical Performance Battery (SPPB), including gait speed. Arterial stiffness parameters were also examined. Questionnaires assessing psychosocial wellbeing were filled in with staff. IG and PG received one comparable exercise session a week lasting 45 minutes for 25-weeks. CON received no intervention. Measurements were performed before and after the intervention.

**Results:**

In children: IG improved all measured physical parameters. When adjusted for baseline values, large effects were observed in favor of IG compared to CON in TGMD-2 (Cohen’s *d*=0.78 [0.33;1.24]) and in handgrip strength (*d* = 1.07 [0.63;1.51]). No relevant differences were found in KOMPIK between groups (−0.38<*d*≤0.14). In seniors: IG showed moderate to very large improvements in all main physical performance (0.61<*d*≤2.53) and psychosocial parameters (0.89<*d*≤1.20) compared to CON.

**Conclusion:**

IG children showed large benefits in motor skills compared to CON while IG seniors benefit especially in psychosocial wellbeing and functional mobility necessary for everyday life. Intergenerational exercise is comparable and in certain dimensions superior to peer group exercise and a promising strategy to integratively improve mental health as well as physical fitness in preschool children and residential seniors.

## Introduction

Throughout the human evolutionary process, intergenerational learning has been the informal vehicle within families to systematically transfer information, knowledge, skills and culture between generations (Hoff, 2007). The last quarter of the 20th century has brought forth demographic and social changes which disrupted the nuclear family and thus severed the generational ties (Newman & Hatton-Yeo, 2008). Intergenerational approaches have been made in social, musical and artistic settings with promising preliminary outcomes in terms of mutual understanding, tolerance and social belonging (Ganss & Narr, 2010; Greger, 2001; Miedaner, 2001; Schmidt & Tippelt, 2009). Although the potential of an intergenerational exercise setting to promote physical as well as psychosocial health in children and seniors has been postulated nearly a decade ago (Granacher et al., 2011a; Granacher et al., 2011b), it has remained unexamined. The combination of the youngest with the oldest of society seems compatible from a variety of standpoints, as they demonstrate similarities in physical predispositions such as net balance and strength performance (Granacher et al., 2011b) as well as mutual needs concerning social learning (Hatton-Yeo, 2007).

Preschool children are in an active neuromuscular developmental process which affects their postural control and muscular strength. In order to support their biological maturation in the best possible way, exercise and varied movement patterns based on fundamental movement skills have been proven to be beneficial, if not mandatory, for healthy childhood development (Barbieri & Zaccagni, 2013; Sibley & Etnier, 2003). Childhood play can not only improve motor skill competence and, in turn, physical performance (Collard et al., 2010), but has also been proven to be a determining factor of long-term fitness, cardiovascular health and participation in recreational as well as elite sports (Lloyd et al., 2015; Vlahov, Baghurst & Mwavita, 2014). Additionally, childhood play and physical activity supports social-emotional and cognitive development through promoting language and communication skills, by improving self-control and memory as well as the ability to cooperate and by teaching problem-solving strategies (De Bock, 2012; Hsieh et al., 2017; Lin et al., 2021).

Seniors show similar values in postural control and muscular strength as children (Granacher et al., 2011a), but in the elderly this state arises from a decrease in neuromuscular performance (Naro et al., 2019) which leads to a loss in postural control and muscular strength (Terao et al., 1996). The ageing process and increasing inactivity impacts not only the neuromuscular, but also the cardiovascular system. Changes in structure and mechanical properties of the vascular bed lead to the degeneration of the arterial wall and an increase in arterial stiffness (Baulmann et al., 2010; Laina, Stellos & Stamatelopoulos, 2018), which manifests itself in systolic hypertension (McEniery et al., 2005). Cognitive changes which occur at older age are primarily influenced by environmental factors and lifestyle along with functional changes due to biological effects (Byun & Kang, 2016) and can be linked to a decrease in physical and social activities (Byun & Kang, 2016). As a result, quality of life starts to decline, independent living cannot be sustained, social interactions are diminished and the occurrence of chronic diseases negatively impact daily activities and overall health (Byun & Kang, 2016; Hollamby, Davelaar & Cadar, 2017; Markle-Reid et al., 2016; Schneider et al., 2017). Both long- and short-term research suggest that physical activity and fitness training in the elderly influence brain structure and function positively (Dishman et al., 2006), thus combating the aforementioned neurological disorders.

As both age groups have similar physical and psychosocial needs, an intergenerational approach combining muscular function and motor skills appears to be not only an innovative strategy to increase physical performance and health in both generations, but also to support the development of children’s social-emotional needs and to improve psychosocial wellbeing and quality of life in seniors. Preliminary data indicates that intergenerational relationships influence participants of exercise programs by providing motivation for seniors to exercise more regularly when children are included and to increase the seniors’ self-esteem (Friedmann & Francine Godfrey, 2007). Nevertheless, the potential and mutually beneficial effects of such exercise settings have not yet been examined.

The Generations on the Move Study therefore aims at examining the physical and psychosocial effects of an intergenerational exercise training intervention compared to peer group training and control settings in preschool children and residential seniors. We hypothesized that, in an intergenerational approach, both age groups improve health-related physical outcomes more than inactive controls and similar to children and seniors who are active within a peer group. We further hypothesized that improvements in psychosocial parameters are larger in the intergenerational group compared to peer group exercisers and controls.

## Methods

### Study design and population

The present study was designed as a five-armed non-randomized controlled trial with a 25-week physical exercise training intervention and pre- and post-intervention assessments. Six kindergartens and five homes for the elderly in Basel-Stadt and surroundings were recruited for the study. The kindergartens and senior homes were assigned either to the intergenerational group (IG), the peer group (PG) or control group (CON). Kindergartens were assigned based on (a) number of children; (b) age of children; (c) migration background of children and; (d) proximity to senior residence homes. Residence homes were allocated based on (a) number of senior participants; (b) age of senior participants and; (c) geographical location of the residence. Children between the ages of 4 and 6 attending the chosen kindergartens without congenital heart defects or any acute diseases were included. Seniors living in the recruited facilities of at least 65 years of age who did not suffer from chronic and/or congenital heart failure, peripheral neuropathy, peripheral arterial occlusive diseases or diabetes mellitus were included. All measurements were non-invasive and performed in the corresponding kindergartens or senior homes. The a priori registered study protocol complies with the ethical standards of the Declaration of Helsinki and was approved by the local ethics committee (Ethikkommission Nordwest- und Zentralschweiz, ethical approval number: 2018-01123; Clinical Trials Registry Identifier: NCT03739385). Seniors and parents of the children signed a written informed consent after receiving all study information.

### Intervention

The intervention groups (IG and PG) received a total of 25 weekly exercise sessions lasting 45 min each. As kindergartens and nursing homes have full schedules, 1 session per week was the only feasible option, which also reflects real-life application of the program. The intervention period was distributed over a scholastic year, taking kindergarten holidays into account. The intervention started in autumn, allowing first year preschoolers the necessary time to adjust to the kindergarten as well as their teachers to observe their social-emotional skills.

The intervention consisted mainly of dynamic balance exercises (walking forwards, backwards, sideways, over objects such as ropes or instable surfaces) as well as object control skills such as throwing, aiming, rolling and catching a variety of objects. Additionally, everyday movement patterns for seniors such as sitting down, standing up, or bending to the floor to pick up objects were integrated in the lessons. Movement patterns for children additionally included jumping, hopping and rolling. The focus during all training sessions were the social interactions between participants, either between peers or between seniors and children. Therefore, the exercises were performed in a playful manner, usually in pairs of two or as a whole group. The intervention did not aim at endurance or cardiovascular strain, as it was based on functional movement patterns. All exercise training sessions were conducted by professional exercise coaches and planned in a progressive and variable manner. In order to compare physical activity between groups, all participants in the intervention groups (20 IG children, 16 IG seniors, 26 PG children and 19 PG seniors) were equipped with accelerometers every five weeks. The data indicates that children showed similar exercise intensities between groups during the training sessions (average over 5 sessions: IG children: 22.8 (1.0) min. moderate-to-vigorous activity (MVPA); PG children: 19.5 (0.6) min. MVPA). IG seniors showed lower MVPA than their corresponding PG (average over 5 sessions: IG seniors: 8.1 (1.0) min. MVPA; PG seniors: 17.9 (1.5) min. MVPA). Even though the intervention was planned so that the overall training stimulus in all intervention groups was equal and therefore comparable, the data showed that the PG seniors were more active than their intergenerational counterparts. This can be explained by the IG performing many exercises while in a seated position. Additionally, the accelerometers were clipped to the participants’ hips and therefore did not register arm or leg movements performed while seated. Nevertheless, all groups performed the same exercises, practiced the same motor skills and followed the same variations and progressions. The intervention was performed in the corresponding institutions, whereby the IG children were accompanied to their partnering home of the elderly for the sessions. Both CON groups received no exercise intervention and were asked to uphold their daily habits.

### Assessment in children

In order to examine the physical as well as social-emotional dimensions, an age-appropriate testing battery including physical performance parameters as well as a questionnaire for social and emotional conduct was applied. We performed all measurements in the respective kindergartens. A team of trained and experienced assessors adhering to standard operation procedures conducted the tests. Kindergarten teachers were included in test standardization for the social-emotional questionnaires. As exercise has a direct effect on hemodynamic measurements, assessments took place on two separate mornings, whereby we assessed motor skill measurements separately from cardiovascular health parameters. This ensured a standardized protocol without interference on the measured parameters.

#### Physical performance

Maximal strength and power were assessed by handgrip strength and counter movement jump (CMJ). Handgrip strength (N) and rate of force development (N/s) of the dominant hand was assessed using the Leonardo Mechanography GF^®^ (Novotec Medical GmbH) device. Children were asked to squeeze the hand grip device as hard as possible for a total of 5s. The CMJ was performed on a force plate (Leonardo Mechanography^®^ GRFP LT). Children were instructed to jump as high as possible and were allowed to use their arms during the jump. Maximal jump power in relation to their body weight (W/kg) and jump height (cm) by flight time were calculated using raw export data. For both tests, the mean values of two valid measurements were used for statistical analysis.

An adapted version of the “Test of Gross Motor Development 2” (TGMD-2) was used to assess gross motor skills (Ulrich, 2000). The test battery is a validated instrument with high test-retest reliability (*r* = 0.88–0.96) (Hardy et al., 2012). The entire TGMD-2 battery included 12 motor tasks consisting of six locomotion (run, hop, gallop, leap, horizontal jump and slide) and six object control subtests (stationary dribble, catch, kick, overhand and underhand throw and striking a stationary ball). As sliding, striking and the underhand throw are strongly linked to American sport culture (Morgan et al., 2013), those three subtests were not included in our testing battery. Each child performed one familiarization trial and subsequently two rated trials for each of the five locomotor skills and four object control skills. Each category was rated according to a dichotomic scale, rating “0” (fail) or “1” (pass) for each of the criteria. A maximum of 56 points could be achieved for locomotor skills, 22 for object control skills and 78 points for the total TGMD-2 score.

#### Social-emotional skills

Kindergarten teachers filled out the KOMPIK questionnaire (skills and interests of children questionnaire) for each child before and after the intervention period to assess social-emotional competence. Three dimensions of the validated questionnaire (Mayr & Bauer, 2014) were applied for the study: (a) social skills (self-assertion and cooperation), (b) emotional skills (empathy and emotional regulation) as well as (c) wellbeing and social relationships. Each dimension consists of four to seven questions to a child’s behavior which were rated with the following scale: (1) “Never/does not apply”; (2) “Rarely”; (3) “Sometimes”; (4) “Mostly/often” and; (5) “Always/very often”. On the basis of the individual answers, scores for each dimension and the total score were calculated. Higher scores represent higher developed skills. Maximum scores for social skills are 70 points, 50 points for emotional skills and 55 points for psychological wellbeing and social relationships which results in a total of 175 possible points as KOMPIK total score (Mayr & Bauer, 2014).

### Assessment in seniors

In order to measure physical as well as psychosocial health, we designed a testing battery consisting of validated, vastly applied clinical screening tools for the elderly, which are indicative for everyday functionality and health. We conducted all measurements in the respective nursing homes. A team of experienced assessors adhering to standard operation procedures conducted the tests. Nursing home staff was included in the test standardization for the questionnaires. All measurements were performed in the morning and the patients were asked to refrain from physical exercise, as well as drinking caffeine or alcohol 12h prior to testing, as these factors can influence cardiovascular outcomes. Patients were furthermore asked to refrain from taking any hypertensive medication the day prior to the measurement. As physical activity has a direct effect on hemodynamic measurements, the cardiovascular measurements were always performed prior to the other physical performance measurements. This ensured a standardized protocol without interference on any parameters.

#### Physical performance

Functional mobility was assessed using the three sub-tests (balance, gait and repeated chair rising test) of the Short Physical Performance Battery (SPPB), a reliable and validated protocol to objectively measure physical performance of the lower extremities in order to identify individuals at risk of poor lower-body function (Guralnik et al., 1994; Volpato et al., 2011). All tests were performed without the use of walking aids such as canes, walking frames or another person for support.

For the balance test, three different stances had to be held for a total of 10s each: Side-by-side, semi-tandem, and tandem stance. A maximum of 4 points can be achieved in the balance sub-test, whereby the scores are attributed as (a) 0 points: not able to hold the stance for 10s or not attempting the stance; (b) 1 point: holding the side-by-side for 10s; holding the semi-tandem stance for 10s; holding the tandem stance between 3 and 9.99s and; (c) 2 points: holding the tandem stance for the entire 10s (Guralnik et al., 1994).

For gait speed, time over 10 m was recorded with timing gates (Witty System) in a single- as well as dual-task situation from which average gait speed was calculated (m/sec.), whereby a split time at 4 m was additionally recorded. Seniors were asked to walk at their habitual walking speed. During dual-task, seniors counted backwards out loud, starting from 50 in the first and from 70 in the second trial. Each walking condition was performed twice and mean values of the tests were used for statistical analysis. The 4 m split time during single-task condition was used for scoring according to cut-off times of the SPPB protocol: 0 points for not being able to walk 4 m distance; 1 point: >8.7s;  2 points: 6.21–8.7s; 3 points: 4.82–6.20s; 4 points: ≤4.82s (Guralnik et al., 1994).

The repeated chair rising test (CRT) is a well-established geriatric test to assess lower-limb power and functionality (Hellmers et al., 2019) and was performed on a force plate (Leonardo Mechanography^®^ GRFP LT) with a 46 cm high locked bench. The force plate records time per repetition as well maximal power during every stand-to-sit cycle. Participants were asked to fully stand up and sit back down as fast as possible without pushing off the bench with their hands. Total time for 5 repetitions (seconds) as well as relative peak power (W/kg) were calculated using raw data exports. The mean of two valid measurements were used for statistical analysis. The total time for 5 sit-to-stand cycles were additionally scored between 1 and 4 points according to SPPB protocol: 1 point: >16.7s; 2 points: 13.7–16.69s; 3 points: 11.2–13.69s; 4 points: ≤11.19s (Guralnik et al., 1994).

These three sub-tests are used to calculate the SPPB total score (maximum score of 12 points), which is highly predictive for disability, hospitalization, institutionalization and mortality in community-dwelling elderly, whereby lower scores indicate higher level of disability (Volpato et al., 2011).

#### Cardiovascular health

Blood pressure and arterial stiffness parameters were obtained using an oscillometric Mobil-O-Graph^®^ PWA Monitor device (I.E.M. GmbH, Stoberg, Germany) with integrated ARCSolver^®^ software. From the measurements, central blood pressure as well the augmentation index corrected for 75 bpm (AiX@75) and pulse wave velocity (PWV) were exported. The blood pressure cuff was placed on the left upper arm while the patient was in a resting seated position. Three valid measurements were taken and the mean values of all three tests calculated and used for data analysis.

#### Psychosocial wellbeing and quality of life

Participants filled out three questionnaires with the help of the research and nursing staff: the general health questionnaire (SF-36), the assessment of quality of life 8 dimensions (AQoL-8D) as well as the fear of falling questionnaire (FES). The SF-36 is a validated and reliable questionnaire consisting of eight categories yielding a summary on physical as well as mental health (Ware Jr, 2000). A higher score in all categories represents higher general health. The AQoL-8D is a reliable and valid tool to assess quality of life (Richardson et al., 2014) in seniors. The questionnaire examines the following dimensions in a total of 35 items: independent living, pain, mental health, life satisfaction, coping, relationships, self-worth and senses. The “senses” dimension was not integrated in the applied questionnaire. The lower the sum in all dimensions, the better is the psychosocial state of an individual. The FES questionnaire consists of 16 questions and is used to assess probability of falling in the elderly (Yardley et al., 2005). The higher the score (range: 16–64), the more severe is the concern about falling.

#### Statistical analysis

Data of all groups is shown as mean with standard deviations (SD). Mean differences in pre-post data for each study arm was calculated with paired t-Tests corresponding 95% confidence intervals (95% CI). Confidence intervals for the pre-post changes in each study arm were estimated by bootstrapping with 5000 re-samples. This analysis was performed by means of estimationstats.com (Ho et al., 2019). Additionally, in order to establish the effects of the intervention groups (IG and PG) compared to control conditions (CON), linear regression models were calculated for each parameter with the respective CON group as model reference. In all analysis, pre-test data as well as age was used as covariate in order to adjust for baseline values. Using the estimates and SD of baseline parameters, effect sizes (Cohen’s *d*) with 95% confidence intervals were calculated and can be interpreted as trivial (*d*<0.2), small (0.2≤*d*<0.5), moderate (0.5≤*d*<0.8) and large (*d*≥0.8) (Cohen, 1992).

## Results

### Study population

**Figure 1:**
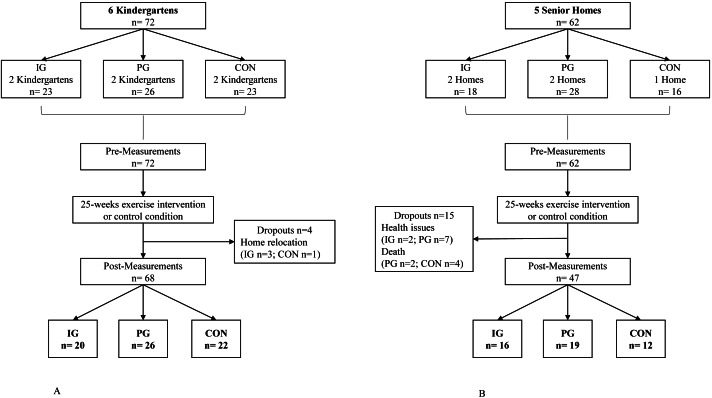
(A) Flow Chart of the study for child participants; (B) Flow Chart of the study for senior participants.

A total of six kindergartens including 72 children and five homes for the elderly with a total of 62 seniors participated in the study. During the study period, four children (6%) and 15 seniors (24%) dropped out due either to home relocation, personal health issues or death. This resulted in a total of 68 children who completed the trial, whereof 20 were in the intergenerational group (IG), 26 in the peer group (PG), and 23 in the control group (CON). A total of 47 seniors completed the study, whereof 16 were in IG, 19 in PG and 12 in CON (Fig. 1). Baseline data for both populations are shown in Table 1.

**Table 1:** Baseline data for children and senior participants for all groups.

	**IG** **Children** ***n* = 20**	**PG** **Children** ***n* = 26**	**CON** **Children** ***n* = 22**	**IG** **Seniors** ***n* = 16**	**PG** **Seniors** ***n* = 19**	**CON** **Seniors** ***n* = 12**
**Gender [f/m]**	11/9	12/14	12/10	15/1	13/6	10/2
**Age (y)**	4.8 (0.8)	4.9 (0.8)	5.0 (0.7)	83.9 (8.2)	80.1 (6.5)	81.4 (6.4)
**Height (cm)**	110.0 (6.4)	113.0 (6.5)	113.0 (5.1)	158.0 (5.8)	165.0 (8.3)	160.5 (6.6)
**Weight (kg)**	18.9 (2.7)	19.8 (2.5)	20.7 (3.8)	63.5 (4.9)	75.2 (14.2)	71.7 (11.1)
**BMI (kg/m**^**2**^)	15.6 (1.2)	15.5 (0.7)	16.0 (1.7)	25.5 (4.9)	27.6 (4.2)	27.7 (3.3)

**Notes.**

Data are reported as means with standard deviation (SD).

IG intergenerational group PG peer group CON control group f female m male BMI body mass index

### Children

#### Physical performance

**Figure 2:**
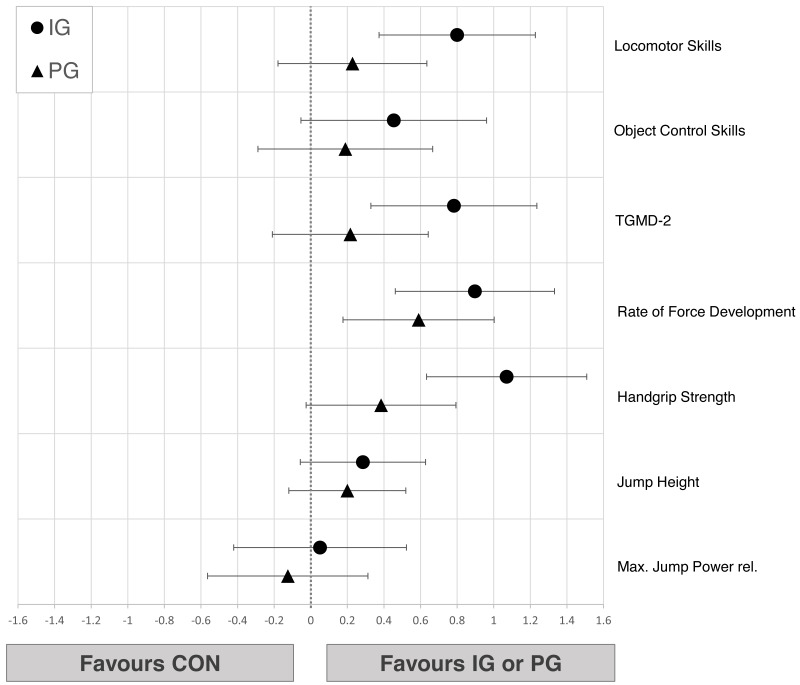
Effects of the intergenerational (IG) and peer groups (PG) on physical performance parameters in children compared to control condition (CON), corrected for baseline values and age. Data are presented as mean between-group differences with 95% confidence intervals.

Changes from pre- to post-test in physical performance parameters of children from all groups are shown in Table 2. Large pre-post differences for IG were found in maximum jump power, handgrip strength and total TGMD-2 score. Linear regression models showed at least moderate improvements of IG compared to CON in most main parameters (0.45<*d* ≤ 1.07). Object control (*d* = 0.45 [−0.05;0.96]) and jump height (*d* = 0.29 [−0.06;0.63]) showed only small improvements in IG relative to CON and a negligible effect in jump power (*d* = 0.11 [−0.30;0.51]). Due to the wide confidence intervals, no explicit statements can be made for PG compared to IG or CON (Fig. 2).

**Table 2:** Physical performance parameters of children.

		**Pre** **Mean (SD)**	**Post** **Mean (SD)**	**Mean Difference (95% CI)**		**Cohen’s*d*** **[95% CI]**	
**Jump height (cm)**	**IG**	13.2 (4.1)	16.6 (3.0)	3.47	[2.03; 4.79]	0.97	[0.49; 1.45]
	**PG**	14.6 (4.5)	16.6 (3.1)	2.02	[0.50; 3.77]	0.53	[0.10; 0.96]
	**CON**	14.3 (4.3)	15.5 (3.5)	1.19	[−2.49; 2.83]	0.23	[−0.42; 0.72]
**Maximum Jump Power (W/kg)**	**IG**	23.4 (3.9)	29.4 (4.7)	5.96	[3.65; 8.56]	1.38	[0.57; 2.04]
	**PG**	27.5 (5.4)	30.2 (4.2)	2.73	[1.10; 4.57]	0.57	[0.18; 0.98]
	**CON**	25.9 (4.8)	30.2 (3.9)	4.29	[3.05; 6.01]	0.98	[0.61; 1.46]
**Handgrip strength (N)**	**IG**	76.9 (16.0)	95.5 (19.2)	18.20	[13.60; 24.10]	1.03	[0.76; 1.28]
	**PG**	83.3 (21.9)	88.2 (21.9)	4.44	[−0.80; 10.40]	0.20	[−0.06; 0.46]
	**CON**	80.7 (17.6)	78.7 (23.3)	−2.46	[−8.99; 1.95]	−0.12	[−0.42; 0.11]
**Rate of force development (N/s)**	**IG**	25.6 (14.8)	36.9 (10.1)	10.90	[3.76; 16.50]	0.86	[0.09; 1.46]
	**PG**	34.7 (16.2)	38.1 (17.7)	2.91	[−0.62; 6.43]	0.17	[−0.05; 0.39]
	**CON**	28.1 (14.8)	24.6 (14.8)	−4.06	[−9.30; 0.56]	−0.27	[−0.57; 0.11]
**Locomotor skills**	**IG**	32.6 (11.0)	40.9 (9.2)	8.30	[4.80; 11.20]	0.82	[0.42; 1.29]
	**PG**	34.5 (7.0)	36.3 (6.1)	1.85	[−1.08; 4.69]	0.28	[−0.18; 0.77]
	**CON**	29.6 (10.1)	32.0 (7.6)	2.45	[−1.43; 6.55]	0.27	[−0.18; 0.76]
**Object control skills**	**IG**	15.2 (5.9)	21.1 (5.9)	5.90	[3.70; 8.25]	1.00	[0.49; 1.60]
	**PG**	15.0 (7.3)	19.2 (6.8)	4.19	[1.88; 6.42]	0.60	[0.26; 0.98]
	**CON**	18.2 (6.6)	19.6 (6.5)	1.41	[−1.68; 4.09]	0.22	[−0.27; 0.70]
**TGMD-2 total score**	**IG**	47.8 (14.1)	62.0 (14.2)	13.90	[9.71; 17.40]	1.00	[0.57; 1.55]
	**PG**	49.5 (12.0)	55.5 (10.9)	6.04	[1.65; 10.20]	0.53	[0.12; 0.94]
	**CON**	47.8 (14.4)	51.7 (12.7)	3.86	[−0.55; 9.32]	0.32	[−0.06; 0.69]

**Notes.**

Data are reported as mean with standard deviation (SD). Mean differences calculated for each group with corresponding effect sizes and 95% confidence intervals [95% CI].

TGMD-2, Test of Gross Motor Skills 2.

#### Social-emotional skills

**Figure 3:**
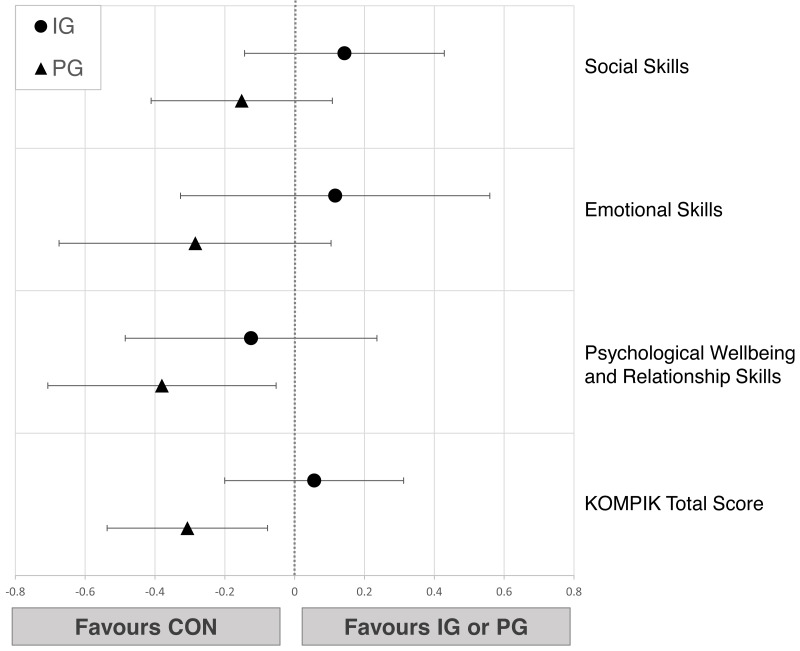
Effects of the intergenerational (IG) and peer groups (PG) on social-emotional skills in children compared to control condition (CON), corrected for baseline values and age. Data are presented as mean between-group differences with 95% confidence intervals.

Pre-post comparison showed small to moderate improvements in IG (0.36<*d* ≤ 0.71) and CON (0.38<*d* ≤ 0.72) while PG showed only trivial effects (−0.09<*d* ≤ 0.12) in all social-emotional dimensions (Table 3). The linear regression models showed no relevant differences when comparing IG to CON (−0.12<*d* ≤ 0.14). PG was slightly inferior to CON in psychological wellbeing (*d* =  − 0.38 [−0.71; −0.05]) and total KOMPIK score (*d* = − 0.31 [−0.54; −0.08]) (Fig. 3).

**Table 3:** Social-emotional skills of children.

		**Pre** **Mean (SD)**	**Post** **Mean (SD)**	**Mean Difference [95% CI]**		**Cohen’s*d*** **[95% CI]**	
**Social Skills**	**IG**	45.5 (16.7)	54.9 (8.8)	9.40	[5.85; 14.60]	0.71	[0.48; 1.01]
	**PG**	56.5 (10.3)	57.7 (9.3)	1.15	[−1.00; 3.62]	0.12	[−0.11; 0.41]
	**CON**	53.2 (6.6)	57.6 (8.3)	4.41	[1.86; 6.68]	0.59	[0.21; 1.00]
**Emotional Skills**	**IG**	34.6 (10.0)	40.1 (5.2)	5.45	[2.70; 8.70]	0.69	[0.37; 1.08]
	**PG**	41.8 (5.3)	41.3 (7.5)	−0.58	[−2.15; 2.23]	−0.09	[−0.36; 0.35]
	**CON**	40.7 (3.8)	42.4 (5.1)	1.73	[−0.05; 3.41]	0.38	[−0.01; 0.84]
**Psychological Wellbeing and Social Relationships**	**IG**	39.8 (8.8)	43.0 (9.1)	3.20	[0.55; 5.50]	0.36	[0.01; 0.68]
	**PG**	47.0 (7.4)	47.4 (8.4)	0.46	[−0.96; 2.00]	0.05	[−0.11; 0.30]
	**CON**	44.7 (4.9)	48.2 (5.0)	3.55	[2.00; 4.95]	0.72	[0.34; 1.12]
**KOMPIK Total Score**	**IG**	119.9 (34.0)	138.0 (19.5)	18.10	[11.20; 25.90]	0.65	[0.45; 0.92]
	**PG**	145.3 (21.3)	146.4 (22.0)	1.04	[−2.54; 5.27]	0.05	[−0.12; 0.27]
	**CON**	138.6 (12.5)	148.2 (15.5)	9.68	[5.32; 13.60]	0.69	[0.37; 1.04]

**Notes.**

Data are reported as mean with standard deviation (SD). Mean differences calculated for each group with corresponding effect sizes and 95% confidence intervals [95% CI].

### Seniors

#### Physical performance

**Figure 4:**
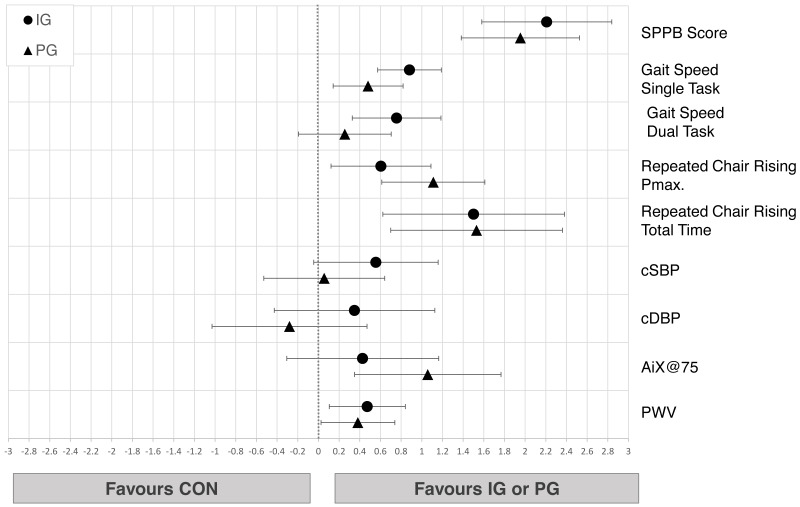
Effects of the intergenerational (IG) and peer groups (PG) on physical performance and cardiovascular health in seniors compared to control condition (CON), corrected for baseline values and age. Data are presented as mean between-group differences with 95% confidence intervals.

Changes from pre- to post-test in physical performance parameters of seniors from all groups are shown in Table 4. We observed small to large decreases in all measured parameters in CON (−0.90<*d*≤-0.36) while IG improved all physical performance variables (0.33<*d* ≤ 1.07) and PG improved all scores (0.62<*d* ≤ 1.10) except gait (−0.23<*d* ≤ 0.06). Linear regressions showed moderate to very large improvements in IG (0.76<*d* ≤ 2.53) and small to very large effects in PG (0.26<*d* ≤ 2.10) compared to CON in all parameters. Regarding differences between IG and PG, the confidence intervals of the effect sizes implicate a compatibility of the data with trivial to at least medium differences in favor of IG for SPPB and for both gait conditions, while trivial to medium effects can be observed for PG in maximum power of CRT (Fig. 4).

**Table 4:** Physical performance and cardiovascular health parameters of seniors.

		**Pre** **Mean (SD)**	**Post** **Mean (SD)**	**Mean Difference** **[95% CI]**		**Cohen’s*d*** **[95% CI]**	
**SPPB Total**	**IG**	6.3 (2.1)	8.0 (2.4)	2.56	[1.69; 3.31]	1.07	[0.61; 1.63]
	**PG**	8.3 (1.1)	9.5 (1.1)	1.32	[0.68; 1.95]	1.10	[0.50; 1.73]
	**CON**	7.8 (2.1)	4.8 (4.0)	−3.0	[−4.42; −1.75]	−0.96	[−1.72; −0.49]
**Rel. Pmax CRT (W/kg)**	**IG**	4.6 (1.5)	5.0 (1.3)	0.45	[0.09; 0.99]	0.33	[0.04; 0.79]
	**PG**	6.2 (1.2)	7.0 (1.3)	0.79	[0.35; 1.20]	0.62	[0.23; 0.98]
	**CON**	4.8 (1.1)	4.0 (1.3)	−0.75	[−1.30; −0.33]	−0.66	[−1.26; −0.26]
**CRT time (s)**	**IG**	19.2 (6.7)	14.4 (8.9)	−4.82	[−8.77; −1.33]	0.61	[−0.04; 1.24]
	**PG**	17.1 (3.5)	13.6 (3.2)	−3.51	[−5.05; −2.33]	1.05	[0.62; 1.60]
	**CON**	17.8 (4.1)	23.4 (9.9)	4.92	[1.97; 9.92]	−0.65	[−1.13; −0.33]
**Gait speed single task (m/s)**	**IG**	0.69 (0.25)	0.88 (0.27)	0.19	[0.12; 0.27]	0.72	[0.42; 1.14]
	**PG**	1.16 (0.24)	1.18 (0.25)	0.01	[−0.04; 0.06]	0.06	[−0.19; 0.26]
	**CON**	0.79 (0.31)	0.68 (0.31)	−0.11	[−0.20; −0.06]	−0.36	[−0.77; −0.16]
**Gait speed dual task (m/s)**	**IG**	0.60 (0.26)	0.72 (0.27)	0.13	[0.07; 0.22]	0.49	[0.18; 0.82]
	**PG**	1.05 (0.26)	0.98 (0.33)	−0.07	[−0.16; 0.01]	−0.23	[−0.58; 0.05]
	**CON**	0.73 (0.30)	0.59 (0.31)	−0.14	[−0.27; −0.05]	−0.46	[−1.09; −0.17]
**cSBP (mmHg)**	**IG**	123 (14)	115 (15)	−8.47	[−16.1; −3.06]	0.58	[0.17; 1.10]
	**PG**	128 (17)	127 (12)	−1.89	[−9.55; 4.92]	0.13	[0.40; 0.66]
	**CON**	122 (24)	126 (17)	3.10	[−17.10;15.9]	−0.15	[−1.00; 0.75]
**cDBP (mmHg)**	**IG**	83 (11)	77 (11)	−6.47	[−11.90; −2.34]	0.58	[0.11; 1.16]
	**PG**	83 (7)	83 (10)	0.26	[−3.32; 4.11]	−0.03	[−0.51; 0.41]
	**CON**	78 (8)	78 (9)	−0.42	[−6.99; 5.08]	0.05	[−0.73; 0.84]
**AIx@75 (%)**	**IG**	31.3 (9.1)	32.6 (11.8)	1.28	[−3.47; 5.03]	−0.12	[−0.55; 0.34]
	**PG**	31.3 (11.8)	25.7 (11.9)	−5.63	[−11.70; −0.82]	0.48	[0.05; 1.00]
	**CON**	28.5 (10.6)	35.6 (7.4)	7.15	[0.90; 13.60]	−0.78	[−1.57; 0.07]
**PWV (m/s)**	**IG**	13.1 (2.1)	13.0 (1.9)	−0.11	[−0.35; 0.08]	0.06	[−0.06; 0.16]
	**PG**	12.5 (1.7)	12.5 (1.4)	−0.02	[−0.35; 0.28]	0.11	[−0.2; 0.26]
	**CON**	12.6 (1.6)	13.3 (1.7)	0.78	[−0.05; 1.73]	−0.48	[−1.02; 0.09]

**Notes.**

Data are reported as mean with standard deviation (SD). Mean differences calculated for each group with corresponding effect sizes and 95% confidence intervals [95% CI].

SPPB Short Physical Performance Battery CRT Repeated Chair Rising Test cSBP central systolic blood pressure cDBP central diastolic blood pressure AIx@75 augmentation index corrected for 75 heartbeats per minute PWV pulse wave velocity

#### Cardiovascular health

Changes from pre- to post-test in cardiovascular parameters of seniors from all groups are shown in Table 4. IG showed decreases of central systolic and diastolic blood pressure while PG showed more favorable effects in lowering AIx@75. Confidence intervals of the linear regressions implicate small differences in favor of IG compared to CON in PWV (*d* = 0.47 [0.10;0.84]). Trivial to small differences between IG and PG in cardiovascular health parameters were observed (Fig. 4).

#### Psychosocial wellbeing and quality of life

**Figure 5:**
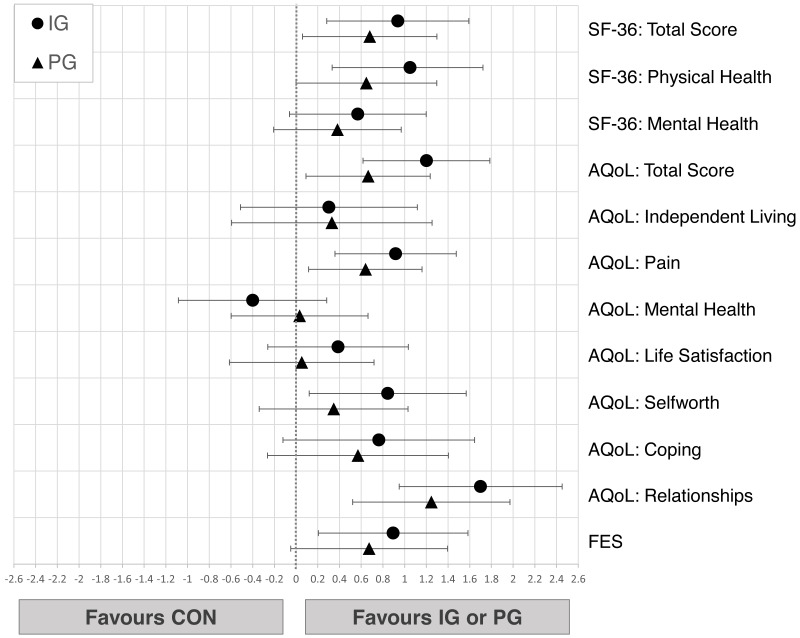
Effects of the intergenerational (IG) and peer groups (PG) on psychosocial wellbeing and quality of life in seniors compared to control condition (CON), corrected for baseline values and age. Data are presented as mean between-group differences with 95% confidence intervals.

Changes from pre- to post-test in psychosocial wellbeing of all groups are shown in Table 5. IG seniors showed small improvements throughout all dimensions of the SF-36 questionnaires (0.31 <*d* ≤ 0.38) while PG showed trivial changes (−0.17<*d* ≤ 0.05) and CON revealed moderate decreases in physical (*d* =  − 0.57) and mental health ( *d* =  − 0.57) (see Table 5). In the AQoL, IG recorded small to moderate improvements in total score (*d* = 0.42), mental health (*d* = 0.53), self-worth (*d* = 0.68) and relationships (*d* = 0.22). PG showed trivial to small decreases (−0.45 <*d* ≤-0.03) while CON revealed small to moderate (−0.77 <*d* ≤ 0.17) declines in all dimensions. Fear of falling (FES) increased in PG (*d* =  − 0.36) and CON (*d* =  − 0.50) while it remained the same in IG (*d* = 0.09). Small to large differences in favor of IG compared to CON are compatible with the data in total SF-36 score (*d* = 0.94 [0.28;1.59]), physical health (*d* = 1.05 [0.33;0.38]), AQoL total score (*d* = 1.20 [0.61;1.79]), pain (*d* =0.92 [0.36;1.48]), relationships (*d* = 1.70 [0.95;2.45]) as well as FES score (*d* = 0.89 [0.21;1.58]). Comparing IG to PG, the observed effects in favor of IG are trivial to small (Fig. 5).

**Table 5:** Psychosocial wellbeing and quality of life of seniors.

		**Pre** **Mean (SD)**	**Post** **Mean (SD)**	**Mean Difference** **[95% CI]**		**Cohen’s*d*** **[95% CI]**	
**SF-36**							
**Total Score**	**IG**	70.5 (20.3)	77.1 (14.6)	6.62	[0.72; 12.80]	0.38	[0.01; 0.71]
	**PG**	78.8 (10.3)	79.4 (14.1)	0.60	[−5.88; 6.88]	0.05	[−0.46; 0.64]
	**CON**	79.5 (12.4)	69.3 (19.9)	−10.20	[−19.50; −4.94]	−0.62	[−1.01; −0.23]
**Physical Health**	**IG**	66.7 (22.4)	74.5 (18.7)	7.77	[0.82; 14.10]	0.37	[0.02; 0.72]
	**PG**	77.4 (12.4)	76.7 (17.0)	−1.15	[−9.13; 5.38]	−0.08	[−0.56; 0.44]
	**CON**	73.3 (15.0)	61.8 (24.1)	−11.5	[−20.60; −3.70]	−0.57	[−1.07; −0.12]
**Mental Health**	**IG**	74.3 (21.2)	79.8 (13.0)	5.47	[−0.97; 14.20]	0.31	[−0.10; 0.77]
	**PG**	84.3 (11.1)	82.2 (12.9)	−2.03	[−6.32; 4.61]	−0.17	[−0.55; 0.44]
	**CON**	85.7 (11.6)	76.9 (18.7)	−8.85	[−18.10; −1.54]	−0.57	[−1.05; −0.01]
**AQoL-8D**							
**Total Score**	**IG**	64.8 (13.3)	59.8 (10.9)	−5.06	[−8.12; −1.94]	0.42	[0.14; 0.82]
	**PG**	55.9 (10.2)	58.4 (12.5)	2.42	[−0.95; 7.11]	−0.21	[−0.55; 0.12]
	**CON**	62.8 (16.3)	73.8 (23.8)	11.00	[5.50; 22.30]	−0.54	[−0.88; −0.30]
**Independent Living**	**IG**	9.4 (2.5)	9.0 (3.5)	−0.44	[−1.38; 0.44]	0.14	[−0.18; 0.52]
	**PG**	6.1 (1.8)	6.7 (2.5)	0.63	[−0.11; 1.42]	−0.30	[−0.64; 0.07]
	**CON**	10.0 (3.4)	10.0 (3.4)	1.58	[−1.25; 3.83]	−0.41	[−1.62; 0.42]
**Pain**	**IG**	4.9 (2.1)	4.9 (2.1)	0.00	[−0.56; 0.81]	0.00	[−0.32; 0.44]
	**PG**	5.3 (2.1)	5.7 (2.0)	0.42	[−0.32; 1.11]	−0.21	[−0.57; 0.17]
	**CON**	6.3 (2.8)	8.0 (3.1)	1.67	[0.50; 2.67]	−0.56	[−1.16; −0.16]
**Mental Health**	**IG**	17.3 (4.6)	15.1 (3.2)	−2.25	[−3.12; −1.44]	0.53	[0.31; 0.78]
	**PG**	15.2 (3.2)	15.6 (3.2)	0.42	[−0.90; 1.53]	−0.13	[−0.48; 0.35]
	**CON**	14.3 (3.7)	14.7 (6.4)	1.67	[0.25; 3.58]	−0.39	[−0.80; 0.00]
**Life satisfaction**	**IG**	8.9 (2.1)	8.6 (2.4)	−0.38	[−1.00; 0.19]	0.17	[−1.33; 0.53]
	**PG**	7.3 (1.3)	8.0 (1.7)	0.68	[−0.05; 1.63]	−0.45	[−0.97; 0.04]
	**CON**	9.3 (3.0)	9.9 (4.0)	0.58	[−0.75; 2.00]	0.17	[−0.53; 0.29]
**Self-worth**	**IG**	6.1 (2.0)	4.9 (1.4)	−1.19	[−1.88; −0.69]	0.68	[0.32; 1.16]
	**PG**	5.2 (1.4)	5.2 (1.8)	0.05	[−0.95; 0.95]	−0.03	[−0.34; 0.67]
	**CON**	5.4 (2.5)	6.1 (3.1)	0.67	[−0.42; 1.92]	−0.24	[−0.70; 0.21]
**Coping**	**IG**	6.3 (1.7)	6.1 (1.5)	−0.25	[−1.00; 0.38]	0.16	[−0.30; 0.64]
	**PG**	5.7 (1.1)	6.1 (1.9)	0.37	[−1.21; 0.32]	−0.24	[−0.74; 0.27]
	**CON**	5.7 (2.3)	7.0 (3.1)	1.33	[0.00; 3.08]	−0.48	[−1.07; 0.04]
**Relationships**	**IG**	11.8 (2.5)	11.3 (2.5)	−0.56	[−1.44; 0.38]	0.22	[−0.20; 0.62]
	**PG**	11.1 (2.4)	11.2 (3.0)	0.11	[−0.58; 1.26]	−0.04	[−0.49; 0.23]
	**CON**	11.8 (3.0)	15.3 (5.7)	3.50	[1.50; 5.50]	−0.77	[−1.37; −0.34]
****							
**FES**	**IG**	25.1 (7.7)	24.3 (8.5)	−0.75	[−3.56; 1.25]	0.09	[−0.17; 0.51]
	**PG**	19.3 (3.7)	21.1 (5.9)	1.79	[0.53; 3.84]	−0.36	[−0.64; −0.12]
	**CON**	27.7 (9.9)	33.4 (12.9)	5.75	[−0.92; 11.80]	−0.50	[−1.03; 0.18]

**Notes.**

Data are reported as mean with standard deviation (SD). Mean differences calculated for each group with corresponding effect sizes and 95% confidence intervals [95% CI].

SF-36 General Health Questionnaire AQoL-8D Assessment of Quality of Life questionnaire, 8 dimensions FES Falls Efficacy Scale

## Discussion

This study examined the effects of an intergenerational exercise training program on physical as well as psychosocial health parameters of preschool children and residential seniors compared to peer group training and inactive control groups. Our results show that both generations, preschool children as well as seniors living in a home for the elderly, can benefit from an intergenerational exercise setting, both on a physical as well as on a psychosocial level. Not only was the intergenerational setting superior to control conditions, the results show that intergenerational exercise is comparable and in certain dimensions superior to peer group exercise.

### Effects in children

Our results show improvements in physical performance in all three groups of children. As childhood is dominated by various biological processes which affect the neuromuscular as well as cognitive development (Beunen , 2000), improvements during the half-year period independently of the intervention were expected. Our data provides two important insights: First, we were able to show that exercise positively influences physical development and should be seen as a crucial element of healthy childhood. Secondly, intergenerational exercise is promising and should be considered as a complementary approach to promote motor development in preschoolers. Compared to both CON and PG, children in IG profit more especially with respect to motor skill development. As studies have shown a strong link between motor skill proficiency and overall long-term physical and cardiovascular fitness (Vlahov, Baghurst & Mwavita, 2014), this finding is of high importance in providing children with a strong foundation for lifelong health. We cannot explain the underlying causes for this development as it was not the scope of our study and should be examined in further research. Nonetheless, certain assumptions can be made. In the intergenerational setting, children were paired with seniors for group activities and exercises. As a consequence, the children had to adapt to their partners in a more distinctive manner than when playing with peers. Seniors move more slowly than children, have longer reaction times and might have certain physical limitations which affect how the children need to interact with them. In order to keep a game ongoing, children might have been compelled to move themselves as well as objects in a more controlled and precise manner, therefore increasing awareness of their movements and demanding better body control. Additionally to the nature of their movement patterns, they might have been in constant motion in order to keep the games ongoing, thus being under a constant, albeit low, load. All groups improved social skills, psychological wellbeing and the ability to build and maintain social relationships. This attests to the notion that childhood is crucial for social-emotional development (Denham et al., 2014). Our data does not allow an interpretation clearly favoring the intergenerational setting. We cannot distinguish whether this is due to the complex nature and synergies which influence social-emotional learning or if the duration and exposure of the intervention was not long enough. The intergenerational setting provides children with many challenges for their social-emotional profile, which do not show short-term effect but might have consequences for the children at a later stage of life. Long-term studies examining this setting are necessary in order to make further statements.

### Effects in seniors

Our physical performance data clearly points towards the importance of regular physical activity in general in older age, especially in residential care settings. Declines in strength, cardiovascular health and functional mobility which occur due to biological aging processes are accelerated by inactivity (Vandervoort, 2002), as shown by our control group. In contrast, both intervention groups were able to not only maintain but increase physical health and functionality parameters. The improvements can be considered relevant, especially since they are a population which, due their living situation, has lost their independence to a notable extent. Both gait speed and SPPB scores are used to assess independent living, hospitalization and mortality in elderly populations (Abellan van Kan et al., 2009; Cabrero-Garcia et al., 2012), and improvements in those scores can have large impacts on individual daily life of such residents by increasing their independence, especially in performing activities of daily life which are based on functional movement patterns. As to the differences between intergenerational and peer groups, one setting does not seem to be superior to the other with regard to physical parameters. Intergenerational seniors improved especially in gait performance and showed lower blood pressure values while seniors in the peer group showed slightly larger gains in maximum power and a larger reduction in arterial stiffness. While both intervention settings benefit, to different degrees, the physical health of seniors, the presence of children has a larger impact on quality of life, perceived mental and physical health as well as on self-worth and relationships of residential seniors. This finding is highly relevant. Studies examining quality of life in elderly institutionalized individuals have shown that quality of life is a complex construct based on internal and external factors. Physical health, social supports and personality traits (Kane, 2003) play an important role in how quality of life is perceived. But quality of life also strongly relates to whether a person is recognized as an individual and by doing meaningful things (Hjaltadottir & Gustafsdottir, 2007). Our results let us assume that the presence and collaboration with children had a direct and positive impact on various dimensions of that construct. A setting which combines functional motor tasks while focusing on interactions between old and young not only increases physical performance but improves psychosocial wellbeing in residential seniors to a larger extent than peer group exercise.

### Methodological considerations

A number of limitations of the study need to be mentioned. Due to the complex setting in kindergartens and senior homes, the participants could not be cluster-randomized in a systematic manner. Nonetheless, it was possible to consider certain criteria when allocating the study arms allowing for balanced baseline data between the age groups. The number of participants per group is relatively small due to the number of study arms and the strict exclusion criteria of senior participants. As this study aimed at examining the effects of a new exercise setting compared to usual exercise settings and control conditions, the number of study arms was necessary and provided valuable information on how to interpret the acquired data. Furthermore, most of our senior participants were female and individuals who enjoy the company of children. Therefore, one must be careful in generalizing and applying the information. Nevertheless, this study reveals important information on two age groups in a field which has not been previously explored and which provides a solid foundation for future studies in the field of intergenerational exercise.

## Conclusion and Practical Applications

In light of demographic, social and economic changes and the direct impact on the relationship between generations (Asheim & Tungodden, 2007), such extra-familiar intergenerational programs possess great potential for both generations. Children profit from intergenerational exercise primarily in motor skill proficiency while seniors benefit, first and foremost, in psychosocial wellbeing while also improving functional mobility necessary for everyday life. Intergenerational exercise is a promising strategy to promote physical performance parameters in children which are necessary for lifelong physical health and fitness while simultaneously challenging their social-emotional learning skills. For seniors, this exercise setting shows great promise in not only maintaining but also improving physical health and especially in benefitting psychological health and elevating quality of life in homes of the elderly. Our study provides necessary evidence proving that the intergenerational exercise setting satisfies the interests and needs of both generations and is, therefore, mutually beneficial. Therefore, opportunities for such exercise should be facilitated. Future large, randomized studies are warranted in order to confirm or refute the observed effects in the intergenerational groups and examine potential underlying causes.

##  Supplemental Information

10.7717/peerj.11292/supp-1 Supplemental Information 1 Complete dataset that was used for statistical analysis, featuring the data described in the methods section

10.7717/peerj.11292/supp-2 Supplemental Information 2 Consort Checklist

## Abbreviations

AIx@75: augmentation index corrected for 75 heartbeats per minute
AQoL-8D: assessment of quality of life questionnaire
BMI: body mass index
cDBP: central diastolic blood pressure
95% CI: 95% confidence intervals
CMJ: counter movement jump
CON: control group
CRT: repeated chair rising test
cSBP: central systolic blood pressure
FES: falls efficacy scale
IG: intergenerational group
MVPA: moderate to vigorous physical activity
PG: peer group
PWV: pulse wave velocity
SD: standard deviation
SF-36: general health questionnaire
SPPB: short physical performance battery
TGMD-2: test of gross motor development, second edition.

## Additional Information and Declarations

### Data Availability

The following information was supplied regarding data availability: The complete dataset is available in the Supplementary File.

### Clinical Trial Registration

The following information was supplied regarding Clinical Trial registration: NCT03739385
